# Clinico-epidemiological and sociodemographic profile of patients with hemophilia in the Brazilian Amazon: High prevalence of hepatitis C infection and its possible corrrelation with inhibitor development

**DOI:** 10.3389/fpubh.2022.963790

**Published:** 2022-09-08

**Authors:** Enzo Miranda Santos, Jean de Melo Silva, Anderson Nogueira Barbosa, Gemilson Soares Pontes

**Affiliations:** ^1^Programa de Pós-Graduação em Hematologia, Universidade Do Estado Do Amazonas (UEA), Manaus, Amazonas, Brazil; ^2^Programa de Pós-Graduação em Imunologia Básica e Aplicada—PPGIBA, Instituto de Ciências Biológicas, Universidade Federal Do Amazonas—UFAM, Manaus, Amazonas, Brazil; ^3^Laboratório de Virologia e e Imunologia, Institituto Nacional de Pesquisa da Amazônia (INPA), Manaus, Amazonas, Brazil

**Keywords:** hemophilia, prevalence, HTLV, HCV, autoantibodies, Amazonas

## Abstract

Hemophilia is a recessive genetic disease caused by a mutation on the X chromosome that has been linked to a high risk of transfusion*-*transmitted infections, especially sexually transmitted infections. The purpose of this retrospective study was to characterize the clinical and epidemiological profile and describe the prevalence of sexually transmitted viral infections in patients with hemophilia in the Northern Brazilian state of Amazonas. We assessed clinical, laboratory and sociodemographic data of hemophiliac patients (*n* = 311) for the period 2011–2019. The majority of the study population was composed of people with a low level of education aged 21–30 years old. The prevalence of HCV, HBV, and HTLV-1/2 infections among the study population were 10.52, 0.52, and 1.05%, respectively. No HIV infection was found among the patients. Between 2011 and 2015 the prevalence of HCV increased by over 100% and the incidence peaked in 2013. The severe hemophilia was associated with the presence of inhibitor factor (Odds Ratio [OD] 9.83; 95% IC: 3.41–27.62, *p* < 0.0001) or target joint (OD 6.59; 95% IC: 3.27–13.34, *p* < 0.0001). The presence of inhibitor was positive and significantly correlated with HCV infection (*r* = 1.00, *p* < 0.0001). Our results showed that HCV infection is highly prevalent in patients with hemophilia and might be involved in the development of inhibitors. Thus, these data provide new insights into the clinical and epidemiological profile of patients suffering from hemophilia in the Northern Brazilian state of Amazonas.

## Introduction

Coagulopathies affect a considerable number of individuals worldwide and hemophilia represents the most common inherited hemorrhagic disorder (1). Hemophilia is a sex-linked disorder, characterized by the impairment of the blood clotting process (2, 3). In some cases, this disorder arises due to a *de novo* mutation (not inherited) and also due to the development of autoantibodies (known as inhibitors) against coagulation factors (1, 4, 5). Hemophilia A is the most frequent subtype, characterized by the deficiency of coagulation factor VIII, while hemophilia B refers to a deficiency of coagulation factor IX (6).

Hemophilia affects ~189,514 people around the world. Hemophilia A occurs in one of every 5,000–10,000 male births, while the prevalence of hemophilia B is one of every 30,000–40,000 male births (7). In absolute numbers, Brazil has the fourth-largest population of patients with hemophilia in the world, which is comprised of 10,821 individuals with hemophilia A and 2,139 with hemophilia B (8, 9). In the Northern Brazilian state of Amazonas, it is estimated that 274 people live with hemophilia A and 37 with hemophilia B (9).

During the 1980s, most hemophiliac patients in Brazil were treated with cryoprecipitate, fresh frozen plasma and plasma-derived clotting factor concentrates. These treatments put patients at high risk of acquiring many blood-borne infectious diseases, such as HIV and hepatitis C (10). Only in 1994, the government of Brazil starts to import the viral inactivated factors VIII (FVIII) and IX (FIX), which resulted in the decrease of blood-transmitted viruses in hemophiliac patients (6, 11). However, the recombinant factor FVIII (rFVIII) became available in Brazil just in 2013 (6). According to the Brazilian Ministry of Health, the prevalence of sexually transmitted infections among people with hemophilia is 1.14% (HIV), 0.97% (HBV), 3.97% (HCV), and 0.1% (HTLV-1/2). However, these numbers are not accurate when prevalence rates are assessed based on Brazilian regions, especially in the north region (12, 13).

The Amazonas is the largest state in Brazil, composed of 62 municipalities, with an estimated population of 4 million people (14). Around 311 patients live with hemophilia in the Amazonas according to the Hematology and Hemotherapy Foundation of Amazonas (HEMOAM). Nevertheless, the clinical epidemiological profile of these patients is unknown. Thus, the purpose of this study was to describe the clinical and epidemiological characteristics and the prevalence of sexually transmitted viral infections in patients living with hemophilia in the Brazilian state of Amazonas.

## Materials and methods

### Ethical aspects

This study was approved by the Human Research Ethics Committee of the Hematology and Hemotherapy Hospital Foundation of Amazonas (approval number: 3.328.121). Confidentiality was assured to all participants. All analyses were performed following relevant guidelines and regulations.

### Study population

This was an observational retrospective study conducted with hemophiliac patients treated at the Hospital Foundation of Hematology and Hemotherapy of Amazonas (HEMOAM). A total of 311 patients with a previous diagnosis of hemophilia A (*n* = 274) and B (*n* = 37) were included. Clinical and socio-demographic information was collected from the hospital's electronic databases. The study population was comprised of patients of either sex and different ethnicities who were aged from 1 to 92 years old.

### Obtention of clinical records

To obtain the clinical data related to viral infections, the patient's clinical records related to the 2011–2019 period were acquired from the Hemovida Web Coagulopathies system. The following information was collected from June 2019 to March 2020: date of birth, date of sample collection, sex, age, results of laboratory tests, comorbidities, treatment, and patient's residence. Patients with incomplete clinical data were excluded from the study.

### Statistical analysis

Descriptive analysis was used to evaluate the total number of patients according to sociodemographic information and the prevalence of viral infections. Prevalence ratio (PR) was used for associations between hemophilia and viral prevalence rates, while the Odds ratio (OR) analysis was used for the associations between the severity of hemophilia and the presence of inhibitor or target joint. OR and PR analyses were done through the chi-square test and Yate's correction with a 95% confidence interval (CI) calculated by the method Koopman asymptotic score and Baptista-Pike, respectively. Spearman's test was applied to assess the correlation between disease severity and the presence of inhibitor, target joint, or positivity for HCV. The correlation was considered strongly positive when *r* > 0.7 All analyzes were performed using the GraphPad Prism v8.0.1 software and values *p* < 0.05 were considered significant.

## Results

### Sociodemographic characteristics of the study population

We analyzed clinical and sociodemographic data of 311 (292 men and 19 women) patients from the northern Brazilian state of Amazonas who have hemophilia. Of those, 88.10% (*n* = 274) had hemophilia A and 11.90% (*n* = 37) hemophilia B. Among individuals with hemophilia A, 95.26% (*n* = 261) were men and 4.73% (*n* = 13) were women, while for hemophilia B, 83.78% (*n* = 31) were man and 16.22% (*n* = 6) were women (Table 1). The mean age observed in patients with hemophilia A was 26.29 ± 15.22 years and 31.49 ± 16.01 years in patients with hemophilia B. The majority of the study population self-identified as brown-skinned (57.56%) and single (57.88%). Regarding the level of education, over 50% of patients did not complete their elementary education. Only 16.08% (*n* = 50) of the study population graduated from high school as the highest education degree (Table 1).

**Table 1:** Sociodemographic profile of hemophiliac patients.

**Sociodemographic characteristics**	***N* (%)**	**Hemophilia A (%)**	**Hemophilia B (%)**
**Gender**			
Male	292 (93.89)	261 (89.38)	31 (10.62)
Female	19 (6.11)	13 (68.42)	6 (31.58)
**Ethnicity**			
Black	27 (8.68)	24 (88.89)	3 (11.11)
White	35 (11.25)	32 (91.43)	3 (8.57)
Brown	179 (57.56)	157 (87.71)	22 (12.29)
Indigenous	27 (8.68)	27 (100.00)	–
No data	43 (13.83)	34 (79.07)	9 (20.93)
**Age**			
1–10	45 (14.47)	41 (91.11)	4 (8.89)
11–20	67 (21.54)	65 (97.01)	2 (2.99)
21–30	87 (27.97)	75 (86.21)	12 (13.79)
31–40	59 (18.97)	49 (83.05)	10 (16.95)
41–50	33 (10.61)	25 (75.76)	8 (24.24)
51–60	11 (3.54)	11 (100.00)	–
>60	9 (2.89)	8 (88.89)	1 (11.11)
**Marital status**			
Single	180 (57.88)	165 (91.67)	15 (8.33)
Married	55 (17.68)	48 (87.27)	7 (12.73)
Consensual union	20 (6.43)	17 (85.00)	3 (15.00)
No data	56 (18.01)	44 (78.57)	12 (21.43)
**Level of schooling**			
Illiterate	14 (4.50)	12 (85.71)	2 (14.29)
Literate	28 (9.00)	27 (96.43)	1 (3.57)
Incomplete middle school	73 (23.47)	71 (97.26)	2 (2.74)
Complete middle school	8 (2.57)	7 (87.50)	1 (12.50)
Incomplete high school	46 (14.79)	43 (93.48)	3 (6.52)
Complete high school	50 (16.08)	42 (84.00)	8 (16.00)
Incomplete undergraduate	8 (2.57)	7 (87.50)	1 (12.50)
Complete undergraduate	17 (5.47)	10 (58.82)	7 (41.18)
Complete post-graduation	3 (0.97)	2 (66.67)	1 (33.33)
No data	64 (20.58)	53 (82.81)	11 (17.19)
**Occupation**			
Student	109 (35.05)	99 (90.83)	10 (9.17)
Retired	7 (2.25)	7 (100.00)	–
Unemployed	3 (0.96)	2 (66.67)	1 (33.33)
Others	94 (30.23)	83 (88.30)	11 (11.70)
No data	98 (31.51)	83 (84.69)	15 (15.31)

**Figure 1:**
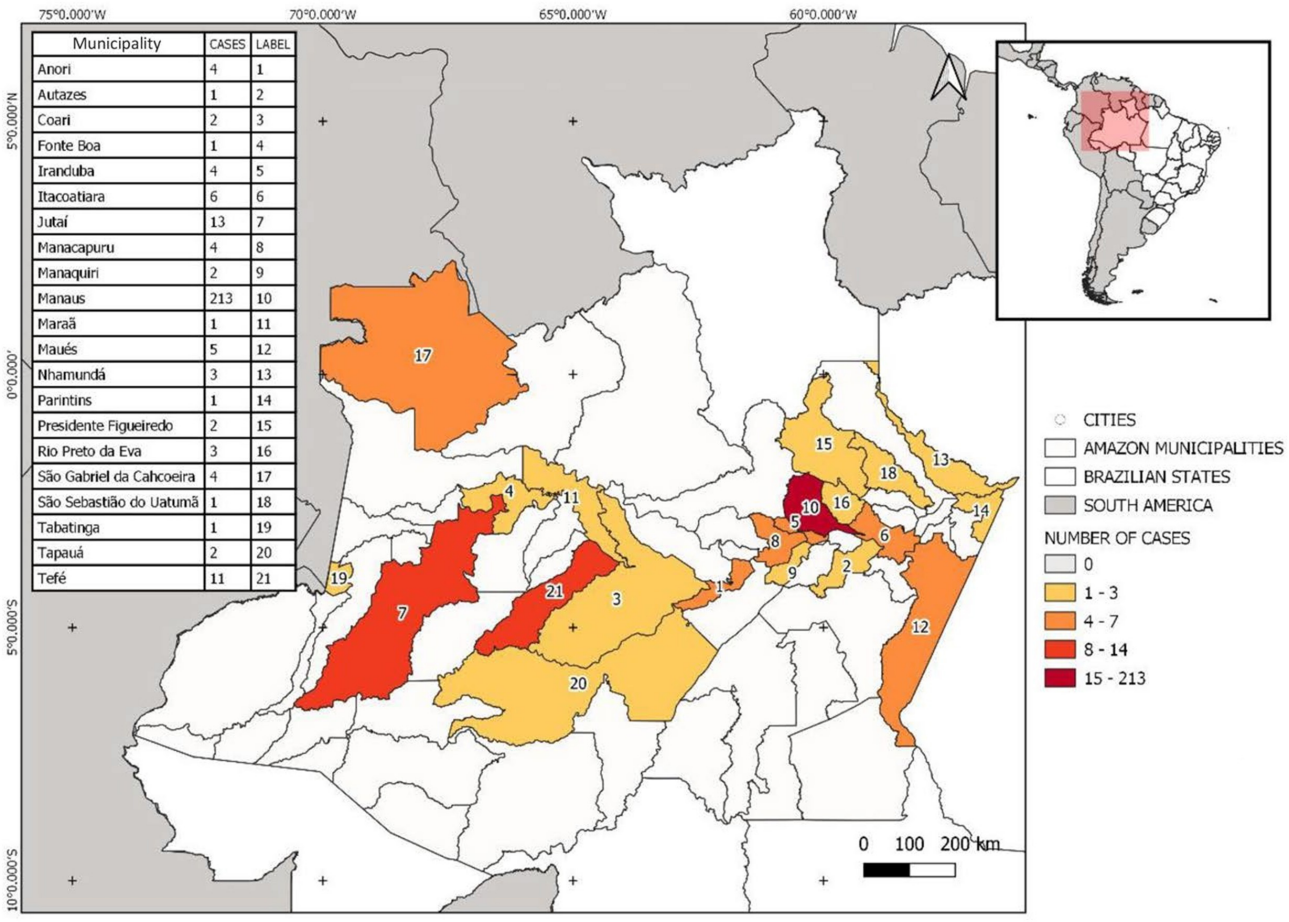
Site map showing the distribution of hemophiliac patients per location within the state of Amazonas. The map was built using the WGS1984 datum (worldclim software v.2.0).

The state of Amazonas consists of 62 municipalities. The study population comprises patients of 21 municipalities, the majority (65%) living in the city of Manaus, the capital of the state of Amazonas. The remaining 35% of the study population resides in small towns situated in the countryside of the state. Jutaí (*n* = 13) and Tefé (*n* = 11) were the municipalities in the countryside with the highest number of hemophiliac patients (Figure 1). No residency information was found for 24 patients.

### Epidemiological profile of blood-borne viruses

**Figure 2:**
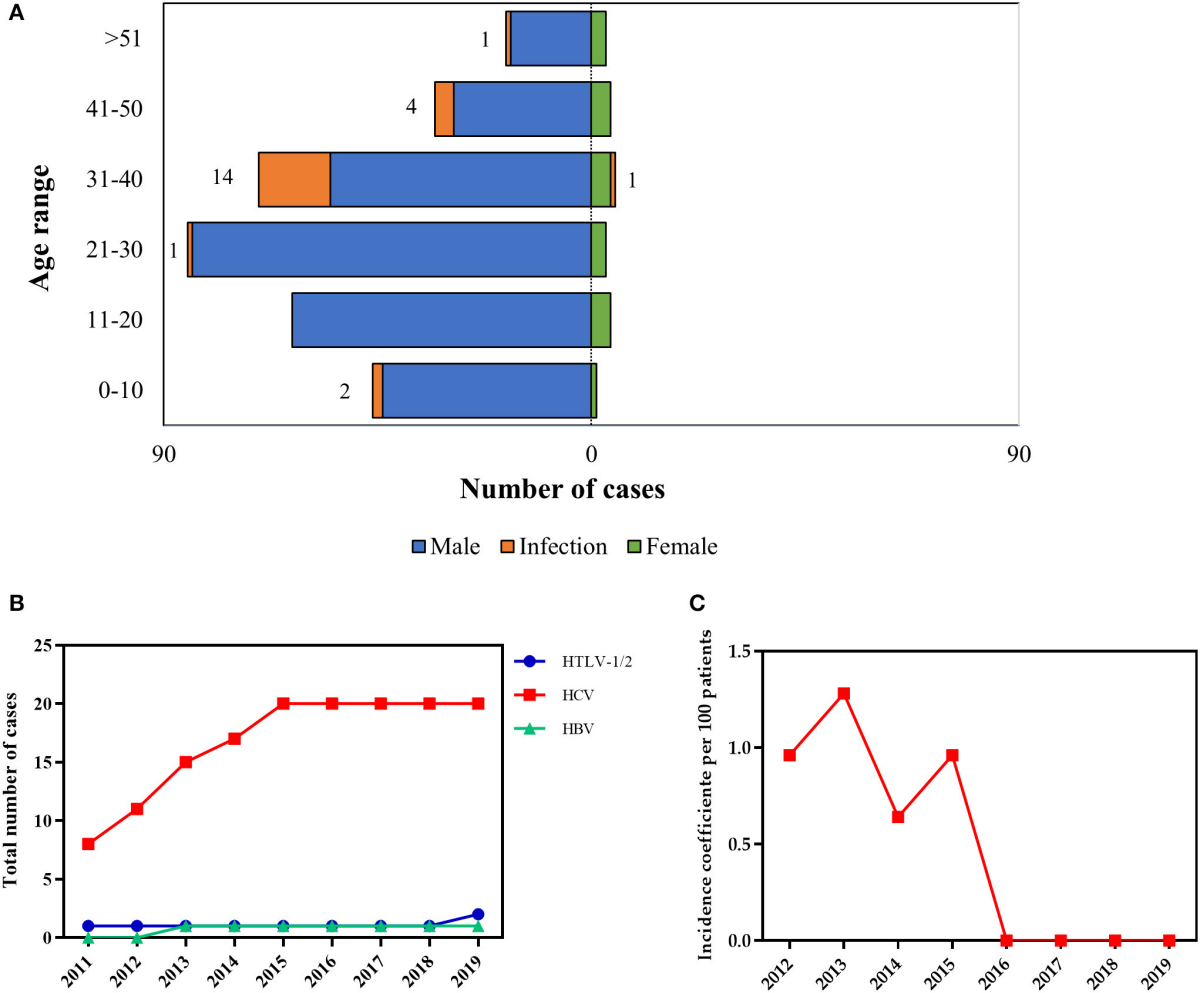
Prevalence and incidence rates of viral infections in patients with hemophilia. **(A)** Numbers of viral infections according to age and sex. **(B)** prevalence HTLV 1/2, HCV, and HBV and **(C)** incidence coefficient of HCV infection according to age.

Only 190 patients had the laboratory records available. Overall, the prevalence of blood-borne viruses was 12.10% (*n* = 23). The majority of those infected were men aged 31–40 years old (*n* = 22) (Figure 2A). Only one woman showed seropositivity for HCV infection, which was the most widespread viral infection (10.45%) in the study population (Supplementary Table 1). Two patients showed seropositivity for HTLV-1/2 (1.05%) and only one patient (0.52%) was positive for HBV infection (laboratory results were positive for anti-HBsAg, anti-HBc and molecular diagnostic). No HIV infection was found in the study population.

Next, we evaluated viral infection prevalence rates from 2011 to 2019 (2020 and 2021 data were not yet available, likely as a result of the coronavirus pandemic). Prevalence rates of HCV infection rose by over 100% between 2011 and 2015, after stabilizing through 2019 (Figure 2B). When we looked at the HCV incidence during the same period, the incidence coefficient (per 100 patients) ranged from 0.68 to 1.28% (Figure 2C). The incidence of HCV infection peaked in 2013.

### Clinical aspects related to hemophilia severity

**Figure 3:**
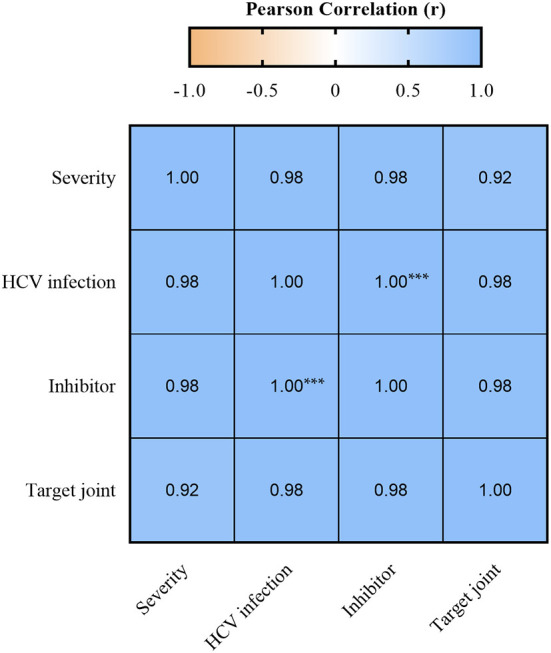
Pearson's correlation between HCV infection, inhibitor, target joint, and severity. Values of *r* > 0.7 were considered strong positive. ***p < 0.0001.

The bivariate analysis carried out to check the correlation between severity, HCV infection, inhibitor and target joint showed that all factors are highly and positively correlated (Figure 3). However, only the correlation between HCV infection and the presence of autoantibodies was statistically significant (*r* = 1.00, *p* < 0.0001). Thus, the presence of HCV infection may be a predisposing factor for the development of inhibitors in the context of hemophilia.

When we looked at the activity of the circulating coagulation factor, we found that 20 patients (7.43%) had autoantibodies (inhibitors), with 16 (80%) of them having severe hemophilia (Table 2). In addition, a total of 46 patients (16.73%) had the clinical condition target joint and 33 (69.57%) of them were suffering from severe hemophilia. The presence of inhibitors was directly associated with severe hemophilia (OD 9.83; 95% IC: 3.41–27.62, p < 0.0001), as was the target joint (OD 6.59; 95% IC: 3.27–13.34, p < 0.0001) (Table 2).

**Table 2:** Association between presence of comorbidities and severity of hemophilia.

**Comorbidities**	**Total *n* (%)**	**Mild**			**Moderate**			**Severe**		
		***n* (%)**	**OR (95% CI)**	***p*-value^a^**	***n* (%)**	**OR (95% CI)**	***p*-value^a^**	***n* (%)**	**OR (95% CI)**	***p*-value^a^**
**Inhibitor (*****n*** **=** **269)**										
Yes	20 (7.43)	1 (5.00)	0.05 (0.005–0.30)	0.0001	3 (15.00)	0.67 (0.20–2.23)	0.3671	16 (80.00)	9.83 (3.41–27.62)	<0.0001
No	249 (92.57)	125 (50.20)	19.15 (3.33–201.60)	0.0001	52 (20.88)	1.50 (0.45–4.97)	0.3671	72 (28.92)	0.10 (0.04–0.29)	<0.0001
**Target joint (***n* **=** **275)**										
Yes	46 (16.73)	2 (4.35)	0.04 (0.01–0.14)	<0.0001	12 (26.09)	1.48 (0.72–3.02)	0.1961	32 (69.57)	6.59 (3.27–13.34)	<0.0001
No	229 (83.27)	126 (55.02)	26.91 (6.97–114.50)	<0.0001	44 (19.21)	0.67 (0.33–1.39)	0.1961	59 (25.77)	0.15 (0.07–0.31)	<0.0001

OR, Odds ratio; CI, Confidence interval calculated by method Baptista-Pike; ^a^Chi-square with Yates' correction.

## Discussion

Epidemiological characteristics of hemophilia in Brazil are still overlooked and poorly described, especially in the Amazonian region. This study outlined epidemiological and clinical aspects of patients suffering from hemophilia in the Northern Brazilian state of Amazonas. Our findings showed that most of the hemophiliac patients in the state of Amazonas consist of unmarried brown-skinned men aged 20–29 years old, with low levels of education and economic status. The frequency of hemophilia observed among women was similar to elsewhere in Brazil and the world (7, 9). The degree of education can influence the clinical prognostic and treatment adherence when associated with other socioeconomic variables (15, 16).

Treatment adherence is a big challenge in the Amazonas state since hemophilia treatment centers exist only in the capital of Amazonas and the majority of small towns in the countryside are at a great distance from Manaus (17). In many cases, long river travel is the only means of transportation available to access health services. As described earlier, 35% of the study population is from hard-to-reach cities, which can undermine the treatment and medical support. This situation may put these patients at high risk of acquiring infectious diseases and developing hemophilia-related comorbidities. However, we found no significant association between these factors and the increase of susceptibility to infectious diseases or the development of comorbidities.

The study population showed an overall prevalence of 12.10% for blood-borne viruses and the most prevalent virus was HCV (10.45%). In Brazil, the prevalence of HCV has been estimated between 0.69 and 1.89%, with a total of 265.815 cases notified in the 1999–2020 period (18–21). Before 1993, HCV and HBV infections were highly prevalent among patients with hemophilia, which dramatically changed after the implementation of mandatory serological and molecular screening during blood donations (6, 13, 22). Nowadays, the estimated seroprevalence for HCV infection in patients with hemophilia is around 3.6% (9). In this study, only one patient was positive for HBV (0.52%), even though the Western Brazilian Amazon is considered highly prevalent for HBV infection (23). The incidence of HCV infection hit its peak in 2013 and the highest prevalence rate was observed in 2015 when the reporting process for hepatitis C cases in Brazil was changed (21). The medical records of the patients did not show any clinical manifestations related to HCV infection, but we emphasize that the laboratory follow-up is not carried out regularly. Considering HCV infection, there is a risk of developing hepatocellular carcinoma in this population, mainly due to the silent evolution (24–28). Our findings also suggest an association between HCV infection and the development of inhibitors.

Some patients with hemophilia A during the administration of FVIII-containing products can develop autoantibodies (inhibitors) against the FVIII factor. The presence of these inhibitors reduces the patient's response to FVIII replacement, resulting in episodes of bleeding that are difficult to control (29). Although the mechanism of inhibitor development in patients with hemophilia is still not completely understood, several factors might be involved such as family history, ethnicity, FVIII gene mutation type, treatment intensity and other unknown environmental factors (30, 31). Our results showed a direct association between positivity for HCV and the presence of inhibitors. Although there are reports of an association between HCV infection and autoantibody development, the present study provides new information on the influence of HCV infection on the development of inhibitors that should be further investigated in more detail (32, 33).

Although our study did not include the patient's genetic aspects or environmental factors, we believe that these factors can influence the severity of the disease, contributing to the development of inhibitors that inactivate the clotting factors. Some studies reported that aspects such as age at the first treatment and intensity of treatment at the first doses are capable of contributing to the worsening of hemophilia (30, 34). Physical aspects can lead to the involvement of some joints. For example, young hemophiliac patients from Taiwan who had a higher degree of obesity were more prone to develop spontaneous joint injuries (35). In this study, we found that the presence of inhibitor and joint target were directly associated with the severity of hemophilia. The factors associated with the presence or development of these comorbidities need further investigation to improve the clinical management of the hemophiliac patient.

HTLV-1/2 infection was found in two patients. Brazil has the largest absolute number of HTLV-1/2 infections in the world and the prevalence varies depending on the region and the population group, ranging from 0.04 to 1% among blood donors (36). North and Northeast regions concentrate the highest rates (37). According to the Ministry of Health, the prevalence of HTLV-1/2 in hemophiliacs is estimated as 0.12% for hemophilia A and 0.10% for hemophilia B (22). Our study showed that the prevalence of HTLV-1/2 (1.14%) in the study population is higher than in the Brazilian population in general. These data indicate that the susceptibility to acquiring HTLV-1/2 infection may be increased in the study population. However, additional studies are required to better clarify the epidemiological aspects of HTLV-1/2 infection among hemophiliac patients in the State of Amazonas.

Although our results propose new data regarding viral infections in hemophiliacs in Amazonas, with results never described before, we understand the limitations of our study, such as the lack of updated epidemiological data, which makes it difficult to obtain complete data. New computer systems have been proposed to organize the sociodemographic data of all patients, especially hemophiliacs (38). The prevalence of HCV and HTLV 1/2 is still above the national average, and further epidemiological studies are needed with a focus on this population to better understand the clinical aspects. Our results demonstrate that some infectious diseases have high prevalence rates. In addition, the absence of routine serological tests is still frequent in the hemophiliac population. Hemophiliac patients in Amazonas have higher prevalence rates for HCV and HTLV 1/2, compared to other population groups, which reveals the need for further studies to provide a better assessment of the hemophiliac population. This study improves the current knowledge on hemophilia epidemiology in the Amazon region, which is important to evaluate factors that can influence the disease prognosis. Also, our findings may help the patient clinical management at the individual and collective levels.

Limitations in this study included the use of secondary data, the possibility of underreporting infections and incorrect records. The quality of monitoring systems varies between regions in Brazil and can have a direct impact on data quality. Generally, underreporting is most pronounced in more vulnerable areas where the access to health services is lower. Furthermore, another limitation is that the diagnosis of HCV is not categorized in the system by the method (serologic or molecular), which makes no distinction between active infections and previous infections.

## Conclusions

In conclusion, this study demonstrated that HCV infection is widespread and could be involved in the induction of inhibitor development in hemophiliac patients of the Amazonas state. The presence of inhibitor or target joints is associated with hemophilia severity. Our findings also draw attention to the specific characteristics of the Amazonian region such as the socio-economic status of the population and the difficulty to reach places, which could impact the treatment adherence and prognosis of hemophilia. Our results enhance the current knowledge on the epidemiology of hemophilia, which is important for assessing factors that may influence the disease's prognosis and may help the clinical management of patients both individually and collectively.

## Data availability statement

The original contributions presented in the study are included in the article/supplementary material, further inquiries can be directed to the corresponding author/s.

## Ethics statement

The studies involving human participants were reviewed and approved by Human Research Ethics Committee of the Hematology and Hemotherapy Hospital Foundation of Amazonas. Written informed consent to participate in this study was provided by the participants' legal guardian/next of kin.

## Author contributions

Conceptualization: GP and ES. Methodology and Investigation: ES, JS, and AB. Validation, data curation, writing-review and editing: GP and AB. Formal analysis and resources: GP, ES, and AB. Writing—original draft preparation: ES and JS. Supervision, project administration and funding acquisition: GP. All authors contributed to the article and approved the submitted version.

## Funding

This study was financed in part by the Coordenação de Aperfeiçoamento de Pessoal de Nível Superior- Brasil (CAPES)- Finance code PROCAD AMAZÔNIA 88881.200581/201801, Pró-Estado Program (#007/2018 and #005/2019) and FAPEAM – POSGRAD 2021.

## Conflict of interest

The authors declare that the research was conducted in the absence of any commercial or financial relationships that could be construed as a potential conflict of interest.

## Publisher's note

All claims expressed in this article are solely those of the authors and do not necessarily represent those of their affiliated organizations, or those of the publisher, the editors and the reviewers. Any product that may be evaluated in this article, or claim that may be made by its manufacturer, is not guaranteed or endorsed by the publisher.
