# Supplementary material for: Clinico-epidemiological and sociodemographic profile of patients with hemophilia in the Brazilian Amazon: High prevalence of hepatitis C infection and its possible corrrelation with inhibitor development
Source: Front Public Health. 2022 Sep 8;10:963790. doi: 10.3389/fpubh.2022.963790 (PMC9493701; doi:10.3389/fpubh.2022.963790)
Supplement: Supplementary file 1 [file Table_1.docx]

**Supplementary Table 1. Prevalence of viral infections.**

| **Viral infections (n=190)** | **Total prevalence N(%)** | **Prevalence according to type of hemophilia (%)** | **PR (95% CI)** | **p-value**^a^ |
| --- | --- | --- | --- | --- |
| HIV | - |  | - | - |
|  |  |  | - | - |
| HCV | 20 (10.52) | A (8.42) | 0.47 (0.19-1.28) | 0.139 |
|  |  | B (2.10) | 2.14 (0.78-5.20) | 0.139 |
| HBV | 1 (0.52) | A (0.52) | - | - |
|  |  | - | - | - |
| HTLV-1/2 | 2 (1.05) | A (1.05) | - | - |
|  |  | - | - | - |

Only individuals with serological tests data were included; PR = Prevalence ratio; CI = Confidence interval calculated by method Koopman asymptotic score; ^a^ Chi-square with Yates' correction; * p<0.05;
